# Establishment and Validation of GV-SAPS II Scoring System for Non-Diabetic Critically Ill Patients

**DOI:** 10.1371/journal.pone.0166085

**Published:** 2016-11-08

**Authors:** Wen-Yue Liu, Shi-Gang Lin, Gui-Qi Zhu, Sven Van Poucke, Martin Braddock, Zhongheng Zhang, Zhi Mao, Fei-Xia Shen, Ming-Hua Zheng

**Affiliations:** 1 Department of Endocrinology, the First Affiliated Hospital of Wenzhou Medical University, Wenzhou, 325000, China; 2 School of the First Clinical Medical Sciences, Wenzhou Medical University, Wenzhou, 325000, China; 3 Department of Hepatology, Liver Research Center, the First Affiliated Hospital of Wenzhou Medical University, Wenzhou, 325000, China; 4 Dept of Anesthesiology, Intensive Care, Emergency Medicine and Pain Therapy, Ziekenhuis Oost-Limburg, Genk, Belgium; 5 Global Medicines Development, AstraZeneca R&D, Loughborough, United Kingdom; 6 Department of Critical Care Medicine, Jinhua Municipal Central Hospital, Jinhua Hospital of Zhejiang University, Jinhua, 321000, China; 7 Department of Critical Care Medicine, Chinese People’s Liberation Army General Hospital, Beijing, China; 8 Institute of Hepatology, Wenzhou Medical University, Wenzhou, 325000, China; Azienda Ospedaliero Universitaria Careggi, ITALY

## Abstract

**Background and Aims:**

Recently, glucose variability (GV) has been reported as an independent risk factor for mortality in non-diabetic critically ill patients. However, GV is not incorporated in any severity scoring system for critically ill patients currently. The aim of this study was to establish and validate a modified Simplified Acute Physiology Score II scoring system (SAPS II), integrated with GV parameters and named GV-SAPS II, specifically for non-diabetic critically ill patients to predict short-term and long-term mortality.

**Methods:**

Training and validation cohorts were exacted from the Multiparameter Intelligent Monitoring in Intensive Care database III version 1.3 (MIMIC-III v1.3). The GV-SAPS II score was constructed by Cox proportional hazard regression analysis and compared with the original SAPS II, Sepsis-related Organ Failure Assessment Score (SOFA) and Elixhauser scoring systems using area under the curve of the receiver operator characteristic (auROC) curve.

**Results:**

4,895 and 5,048 eligible individuals were included in the training and validation cohorts, respectively. The GV-SAPS II score was established with four independent risk factors, including hyperglycemia, hypoglycemia, standard deviation of blood glucose levels (Glu_SD_), and SAPS II score. In the validation cohort, the auROC values of the new scoring system were 0.824 (95% CI: 0.813–0.834, P< 0.001) and 0.738 (95% CI: 0.725–0.750, P< 0.001), respectively for 30 days and 9 months, which were significantly higher than other models used in our study (all P < 0.001). Moreover, Kaplan-Meier plots demonstrated significantly worse outcomes in higher GV-SAPS II score groups both for 30-day and 9-month mortality endpoints (all P< 0.001).

**Conclusions:**

We established and validated a modified prognostic scoring system that integrated glucose variability for non-diabetic critically ill patients, named GV-SAPS II. It demonstrated a superior prognostic capability and may be an optimal scoring system for prognostic evaluation in this patient group.

## Introduction

Critical care medicine is a multi-disciplinary specialty concerned with the management of life-threatening conditions in critically ill patients. These patients account for 11.3% of hospital mortality and even a high mortality rate in the six months after discharge [1,2]. Over the last three decades, several scoring systems for critical illness have been proposed for assisting physicians to quantify severity of disease and assess the prognosis. The Simplified Acute Physiology Score (SAPS) is one of the most widely used scoring systems at intensive care unit (ICU), which was first constructed in 1984 as an improvement of the Acute Physiology And Chronic Health Evaluation (APACHE) scoring system. The second generation SAPS score (SAPS II), was further validated in several studies and proved to be applicable in other cohorts [3,4,5,6].

Blood glucose levels are a crucial physiological variable for patients admitted to an ICU department with infection, sepsis and other critical conditions [7,8,9]. Of note, acute hyperglycemia and hypoglycemia were reported as independent detrimental factors for hospital mortality [10]. In scoring systems for critical illness, however, serum glucose levels have shown no significant association after adjusting for other parameters [6,11]. In recent years, glucose variability has been increasingly recognized as an independent risk factor for mortality in non-diabetic patients at ICU rather than blood glucose level [12,13,14]. Therefore, glucose variability can be considered as a novel parameter in scoring system for non-diabetic subjects at ICU.

The aim of this study was to construct and validate a modified SAPS II scoring system with additional glucose variability parameters. The system is designed to be specific for non-diabetic patients from ICU and was tested on a patient cohort from the Beth Israel Deaconess Medical Center to determine its effectiveness in predicting the accuracy of SAPS II for the risk of short-term and long-term mortality. Furthermore, the prognostic ability of the novel scoring system was compared with other standard scoring systems.

## Material and Methods

### The database

The Multi-parameter Intelligent Monitoring in Intensive Care III version 1.3 (MIMIC-III v1.3) is a publicly and freely available database comprising de-identified health-related data associated with over forty thousand patients who come from a variety critical care units of the Beth Israel Deaconess Medical Center between 2001 and 2012 [15]. In order to apply for permission to access the database, researchers are mandated to complete the NIH web-based training course named “Protecting Human Research Participants” (Our certification number: 1605699).

### Study design

In this study, we by extracted data from MIMIC III database, established and validated a modified SAPS II scoring system incorporating glucose variability and named the new scoring system Glucose Variability associated SAPS II Scoring System (GV-SAPS II). The training cohort consisted of individuals admitted to ICU in the Beth Israel Deaconess Medical Center from 2001 to 2008 and the validation cohort comprised of patients admitted during 2009 to 2012 from the same database.

The start date for follow-up was the date of patient’s admission. The date of death for patients was obtained from Social Security Death Records from the US government. All the patients were followed up for at least 9 months.

### Population selection and definitions

A total of 58,976 ICU admissions were recorded in the MIMIC III database. Patients with diabetes were excluded from our study, and this was determined by medical history, diagnosis at admission (International Classification of Disease 9 code: 250.xx) or admission HbA1c value of ≥ 6.5% (recommended for the diagnosis from the American Diabetes Association [16]). For non-diabetic patients, hyperglycemia was defined as any serum glucose level ≥ 11.1 mmol/l, and hypoglycemia was defined as any glucose measurement ≤ 3.9 mmol/l [17].

Subjects who met the following criteria were excluded: (1) missing of individual data greater than 5% or lack of glucose measurements;(2) outliers exited; (3) diabetic patients; (4) age < 18y; (5) not the first admission; (6) hospital stay length less than 2 days; (7) total glucose measures < 3 times; (8) the mean interval of glucose records more than 24 hours.

### Date extraction

Patient data was exacted from MIMIC III using structure query language (SQL) with Mysql tools (version 5.6.24), including patient identifiers, demographic parameters, clinical parameters, laboratory parameters and scoring systems. According to the patient identifier system, we can obtain the hospital records of a particular patient from 2001 to 2012 at Beth Israel Deaconess Medical Center. Records of baseline characteristics were exacted in the first 24 hours after patient admission.

Physiological information (heart rate, respiratory rate, systolic blood pressure and diastolic blood pressure) was measured by bedside monitors. Age, gender, the length of stay in hospital, readmission records were also recorded in the database.

Laboratory measurements included white blood cell (WBC) and platelet count, urea nitrogen (BUN), serum potassium, serum sodium, partial pressure of oxygen (PO_2_), fraction of inspiration O_2_, bicarbonate, serum glucose, creatinine, and bilirubin. The mean interval of glucose records were 20 hours. Hyperglycemia and hypoglycemia were mapped to classes according to the following thresholds: 0: non-hyperglycemia, non-hypoglycemia; 1: hyperglycemia, hypoglycemia.

Three other standard scoring systems were evaluated enabling a comparison with our GV-SAPS II (original SAPS II, Sepsis-related Organ Failure Assessment Score (SOFA) and Elixhauser comorbidity score). Scores were all calculated using physiological measurements and clinical information according to published recommendations and accepted formulae [6,18,19].

### Construction of the GV-SAPS II Score

In this study, three parameters were defined as glucose variability components and are: hyperglycemia, hypoglycemia and standard deviation of blood glucose levels (Glu_SD_). For the training cohort, glucose variability components and SAPS II score were selected for Cox proportional hazard regression analysis for determining the association with prognosis and survival time. The hazard or instantaneous risk of death h(t) at time t after randomization for a patient with variables x_l_,…,x_n_ has the form *h*(*t*) = *h*_0_(*t*) *exp*(*b*_1_*x*_1_ + *b*_2_*x*_2_ + … *b*_*n*_*x*_*n*_). According to the coefficients, a prognostic index (*PI* = *b*_1_*x*_1_ + *b*_2_*x*_2_ + … + *b*_*n*_*x*_*n*_) can be calculated for each patient on the basis of the final mode. Higher values of index signify a worse prognosis, and lower signify a better prognosis [20]. Therefore, the PI can be used as a novel prognostic scoring system, named GV-SAPS II score, based on four parameters (hyperglycemia, hypoglycemia, Glu_SD_ and SAPS II score). For ease of use, we defined GV-SAPS II score as a ten-fold PI.

To compare the 30-day and 9-month prognostic ability of GV-SAPS II score with other models, the area under the curve of the receiver operator characteristic (auROC) curve was determined, which is a measure of discrimination. In addition, the standard index of validity, such as the Youden index, sensitivity, specificity, positive likelihood ratio, negative likelihood ratio, positive predictive value, and negative predictive value, were calculated according to the ROC results.

### Statistical analysis

We categorized Glu_SD_ into three groups using optimal binning strategies: G1: ≤ 0.7 mmol/l, G2: 0.7 to 2.1 mmol/l, G3: ≥ 2.1 mmol/l. In the training cohort, the hazard ratios (HRs) and 95% confidence intervals (CIs) of scoring system parameters were calculated using Cox proportional hazard regression. In addition, Kaplan–Meier survival curves were calculated to describe the incidence of outcomes after 30 days and 9 months and stratified by different risk levels of the GV-SAPS II.

The Kolmogorov–Smirnov test was used to determine whether sample data were likely to be derived from a normal distribution population. Continuous variables were summarized as mean ± standard deviation (SD) or median (inter-quartile range (IQR)), respectively. The categorical variables were displayed as counts or percentages (%). The characteristics of the study population in two cohorts were compared using Student’s t test or non-parametric Wilcoxon test for continuous variables and χ^2^-test for categorical variables. All P-values were two-sided and a P value of < 0.05 was considered statistically significant. Analyses were performed in SPSS version 20.0 (SPSS, Chicago, IL, USA), MedCalc version 12.7 (MedCalc Software, Ostend, Belgium).

## Results

### Characteristics of the study sample

**Fig 1:**
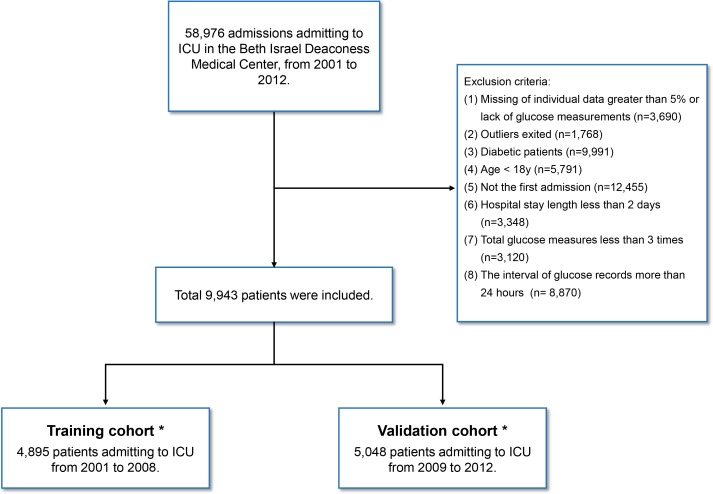
A flow diagram of study participants. *There is no overlap between training and validation cohorts.

A total of 58,976 admission records were extracted and enrolled in our cohort. After exclusion of those who did not meet the inclusion criteria, 4,895 and 5,048 eligible individuals were finally included in the training and validation cohorts, respectively (Fig 1).

Table 1 summarizes the patient characteristics and glucose indices for the two cohorts. In the training cohort, median and IQR of Glu_SD_ was 1.1 mmol/l (0.7 to 1.8 mmol/l), of which the proportion of hyperglycemia patients was 14.2% and hypoglycemia was 6.6%. In the validation cohort, median and IQR of GluSD was 1.0 mmol/l (0.7 to 1.6 mmol/l), of which the proportion of hyperglycemia patients was 10.6% and hypoglycemia was 6.0%. The scoring systems of training subjects showed the scores were 4.0 (2.0 to 6.0), 31.0 (22.0 to 45.0), 0.0 (-1.0 to 4.0) in SOFA, SAPS II, Elixhauser score, respectively. In the validation cohort, the prognostic scores were 3.0 (1.0–5.0), 29.0 (21.0–43.0), 0.0 (-1.0–6.0) in SOFA, SAPS II and Elixhauser score. Hospital characteristics and clinical outcomes showed that the 30-day mortality of subjects was 12.5% and 9.7% in training and validation cohorts, respectively. Furthermore, the mortality of the two cohorts were 18.7% and 15.9% at the end of 9 months.

**Table 1:** Characteristics of critically ill patients in training and validation cohorts.

	Training cohort	Validation cohort	P
	(n = 4895)	(n = 5048)	
**Demographic parameters**			
Age, y	59.7 ± 17.7	60.8 ± 17.4	0.002
Gender, n (%)			0.224
Female	2022 (41.3%)	2146 (42.5%)	
Male	2873 (58.7%)	2902 (57.5%)	
Ethnicity, n (%)			< 0.001
White	3416 (69.8%)	3814 (75.6%)	
Black	280 (5.7%)	366 (7.3%)	
Others	1199 (24.5%)	868 (17.2%)	
**Laboratory parameters**			
Glu_avg_ (mmol/l), median (IQR)	6.4 (5.8–7.2)	6.3 (5.7–7.0)	< 0.001
GluSD (mmol/l), median (IQR)	1.1 (0.7–1.8)	1.0 (0.7–1.6)	< 0.001
Hyperglycemia rate, n (%)	696 (14.2%)	536 (10.6%)	< 0.001
Hypoglycemia rate, n (%)	321 (6.6%)	303 (6.0%)	0.254
**Clinical parameters**			
Mechanical ventilation rate, n (%)	2028 (41.4%)	1897 (37.6%)	< 0.001
Length of hospital stay, d, median (IQR)	4.0 (3.0–5.0)	4.0 (3.0–5.0)	< 0.001
Readmission rate, n (%)	776 (15.9%)	635 (12.6%)	< 0.001
Hospital mortality, n (%)	649 (13.3%)	468 (9.3%)	< 0.001
30-day mortality, n (%)	611 (12.5%)	489 (9.7%)	< 0.001
9-month mortality, n (%)	914 (18.7%)	803 (15.9%)	< 0.001
**Scoring systems**			
GCS, median (IQR)	15.0 (8.0–15.0)	15.0 (8.0–15.0)	0.024
SOFA, median (IQR)	4.0 (2.0–6.0)	3.0 (1.0–5.0)	< 0.001
SAPS II, median (IQR)	31.0 (22.0–45.0)	29.0 (21.0–43.0)	< 0.001
Elixhauser score, median (IQR)	0.0 (-1.0–4.0)	0.0 (-1.0–6.0)	0.096

**NOTE.** Normal distributed data presented as mean ± SD (P < 0.05; independent Student’s t-test); non-normal distributed data presented as median (IQR) (P < 0.05; non-parametric Wilcoxon test); categorical variables presented as counts (n) or percentages (%).

Glu_avg_ = average of glucose levels, Glu_SD_ = standard deviation of glucose levels.

### Construction of the GV-SAPS II

Glucose variability was defined as the Glu_SD_ per patient in our study and this has been widely used in previous studies [21]. Measures of hyperglycemia and hypoglycemia as outcomes of serious glucose fluctuations, were included in the glucose variability components as well.

As shown in Table 2, both glucose variability components (Glu_SD,_ hyperglycemia, hypoglycemia) and SAPS II scores were associated with 30-day mortality of critically ill patients in the univariate analysis. After entering the data into the multivariate Cox proportional hazards regression analyses, these components and the SAPS II score were shown to be independent risk factors for 30-day mortality. The hazard ratio of each variables as follow: Glu_SD_ level in G2 (HR = 1.731, 95% CI: 1.300–2.304, P < 0.001), Glu_SD_ level in G3 (HR = 1.641, 95% CI: 1.100–2.448, P < 0.015), hyperglycemia (HR = 2.516, 95% CI: 1.860–3.405, P < 0.001), hypoglycemia (HR = 1.952, 95% CI: 1.556–2.449, P < 0.001), SAPS II score (HR = 1.055, 95% CI: 1.050–1.060, P < 0.001).

**Table 2:** Univariate and multivariate analysis of the associations between 30-day mortality and Glucose Variables with SAPS II in training cohort.

Variables		Univariable				Multivariable		
	B	HR	95% CI	P	B	HR	95% CI	P
Glu_SD_, reference (G1:< 0.7 mmol/l)	0	1.000	-	-	0	1.000	-	-
Glu_SD_ (G2: 0.7–2.1 mmol/l)	0.812	2.251	1.695–2.991	< 0.001	0.549	1.731	1.300–2.304	< 0.001
Glu_SD_ (G3: > 2.1 mmol/l)	1.849	6.352	4.746–8.501	< 0.001	0.495	1.641	1.100–2.448	0.015
Hyperglycemia*	1.436	4.206	3.571–4.953	< 0.001	0.923	2.516	1.860–3.405	< 0.001
Hypoglycemia*	1.005	2.731	2.186–3.413	< 0.001	0.669	1.952	1.556–2.449	< 0.001
SAPS II	0.062	1.064	1.060–1.069	< 0.001	0.054	1.055	1.050–1.060	< 0.001

**NOTE**. HR, Hazard Ratio; CI, confidence interval; Glu_SD_, standard deviation of blood glucose levels.

*For hyperglycemia, 0: non-hyperglycemia, 1: hyperglycemia; for hypoglycemia, 0: non-hypoglycemia, 1: hypoglycemia

Finally, these four parameters were included in novel GV-SAPS II scoring system. The formula could be calculated by combining their regression coefficients (a tenfold PI):

$$GV-SAPS\;II=B-Glu_{SD}+9.23\;*\;hyperglycemia+6.69\;*\;hypoglycemia+0.54\;*\;SAPS\;II$$

$$\begin{array}{l} B-Glu_{SD}=0,\;Glu_{SD}<\;0.7\;mmol/l;\\ B-Glu_{SD}{}_{}=5.49,\;0.7\;mmol/l\leq Glu_{SD}<\;2.1\;mmol/l;\\ B-Glu_{SD}=4.95,\;Glu_{SD}\geq \;2.1\;mmol/l \end{array}$$

### Application of GV-SAPS II to ICU short-term and long-term outcomes

After applying the GV-SAPS II scoring system for enrolled subjects, the mean scores from the training and validation cohorts were 24 ± 10, and 19 ± 10 respectively. In the training cohorts, the HRs of the new scoring system were 1.098 (95% CI: 1.088–1.107, P < 0.001) and 1.093 (95% CI: 1.085–1.101, P < 0.001) for 30-day and 9-month mortality. In the validation cohort, HRs were 1.111 (95% CI: 1.102–1.121, P < 0.001) and 1.102 (95% CI: 1.093–1.111, P < 0.001) in adjusted models.

**Fig 2:**
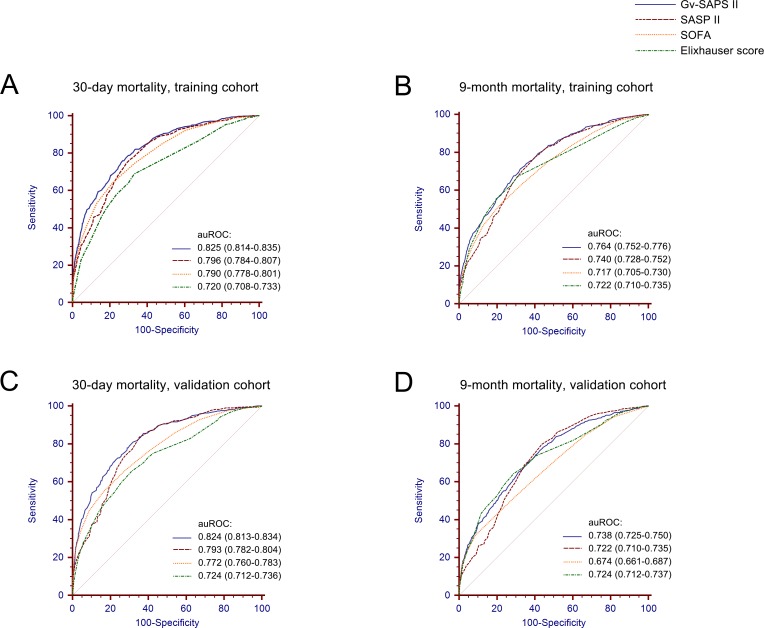
ROC analysis of the prognostic efficiency of GV-SAPS II score and other models to predict short-term and long-term outcomes in training cohort and validation cohort.

The performance of GV-SAPS II to predict 30-day and 9-month outcomes in the training cohort is presented in Fig 2A and 2B using ROC analysis. The auROC of new scoring system was 0.825 (95% CI: 0.814–0.835, P< 0.001) after 30 days and 0.764 (95% CI: 0.752–0.776, P< 0.001) after 9 months, which were significantly higher than other scoring systems determined in our study (all P < 0.001). The auROC of SAPS II, SOFA, Elixhauser scores are: 0.796 (95% CI: 0.784–0.807, P< 0.001), 0.790 (95% CI: 0.778–0.801, P< 0.001), 0.720 (95% CI: 0.708–0.733, P< 0.001) in 30 days and 0.740 (95% CI: 0.728–0.752, P< 0.001), 0.717 (95% CI: 0.705–0.730, P< 0.001), 0.722 (95% CI: 0.710–0.735, P< 0.001) after 9 months. Moreover, we used an optimal cut-off value of 28 and 26 for 30-day and 9-month prediction respectively. The sensitivities were 75.94% and 71.33% respectively, the specificities were 73.23% and 67.55% respectively (Table 3).

**Table 3:** Performance parameters of scoring system as predictors of short-term and long-term mortality of critically ill subjects.

Models	auROC (95%)	P	Cut-Off Point	Se (%)	Sp (%)	+LR	-LR	+PV	-PV
**Training cohort (30-day)**									
GV-SAPS II	0.825 (0.814–0.835)	< 0.001	28	75.94	73.23	2.84	0.33	28.8	95.5
SAPS II	0.796 (0.784–0.807)	< 0.001	38	74.63	71.15	2.59	0.36	27	95.2
SOFA	0.790 (0.778–0.801)	< 0.001	5	64.32	78.41	2.98	0.46	29.8	93.9
Elixhauser score	0.720 (0.708–0.733)	< 0.001	0	68.9	66.9	2.08	0.46	22.9	93.8
**Training cohort (9-month)**									
GV-SAPS II	0.764 (0.752–0.776)	< 0.001	26	71.33	67.55	2.2	0.42	33.5	91.1
SAPS II	0.740 (0.728–0.752)	< 0.001	32	77.46	59.98	1.94	0.38	30.8	92.1
SOFA	0.717 (0.705–0.730)	< 0.001	5	51.86	78.8	2.45	0.61	36	87.7
Elixhauser score	0.722 (0.710–0.735)	< 0.001	0	67.18	69.23	2.18	0.47	33.4	90.2
**Validation cohort (30-day)**									
GV-SAPS II	0.824 (0.813–0.834)	< 0.001	26	77.91	71.11	2.7	0.31	22.4	96.8
SAPS II	0.793 (0.782–0.804)	< 0.001	33	83.64	64.69	2.37	0.25	20.3	97.4
SOFA	0.772 (0.760–0.783)	< 0.001	5	55.01	83.4	3.31	0.54	26.2	94.5
Elixhauser score	0.724 (0.712–0.736)	< 0.001	3	65.44	68.87	2.1	0.5	18.4	94.9
**Validation cohort (9-month)**									
GV-SAPS II	0.738 (0.725–0.750)	< 0.001	24	70.61	63.25	1.92	0.46	26.7	91.9
SAPS II	0.722 (0.710–0.735)	< 0.001	29	79.83	56.28	1.83	0.36	25.7	93.6
SOFA	0.674 (0.661–0.687)	< 0.001	6	31.76	91.33	3.66	0.75	40.9	87.6
Elixhauser score	0.724 (0.712–0.737)	< 0.001	3	64.26	71.19	2.23	0.5	29.7	91.3

**NOTE.** Se, sensitivity; Sp, specificity; +LR, positive likelihood ratio; -LR, negative likelihood ratio; +PV, positive predictive value; -PV, negative predictive value; CI, confidence interval.

In the validation cohort, the novel scoring system also presented an improved capability to predict 30-day and 9-month mortality. As shown in Fig 2C and 2D, the new scores were assessed with an auROC of 0.824 (95% CI: 0.813–0.834, P< 0.001) for 30 days and 0.738 (95% CI: 0.725–0.750, P< 0.001) for 9 months. In the same cohort, SAPS II had an auROC of 0.793 (95% CI: 0.782–0.804, P< 0.001) and 0.722 (95% CI: 0.710–0.735, P< 0.001), SOFA scores of 0.772 (95% CI: 0.760–0.783, P< 0.001) and 0.674 (95% CI: 0.661–0.687, P< 0.001), Elixhauser scores of 0.724 (95% CI: 0.712–0.736, P< 0.001) and 0.724 (95% CI: 0.712–0.737, P< 0.001), significantly lower than GV-SAPS II score (all P < 0.001). Using the best cutoff values of 26 and 24 for 30 days and 9 months, the sensitivities were 77.91% and 70.61% respectively, the specificities were 71.11% and 63.25% respectively (Table 3).

### Survival distributions in different risk levels of the GV-SAPS II

**Fig 3:**
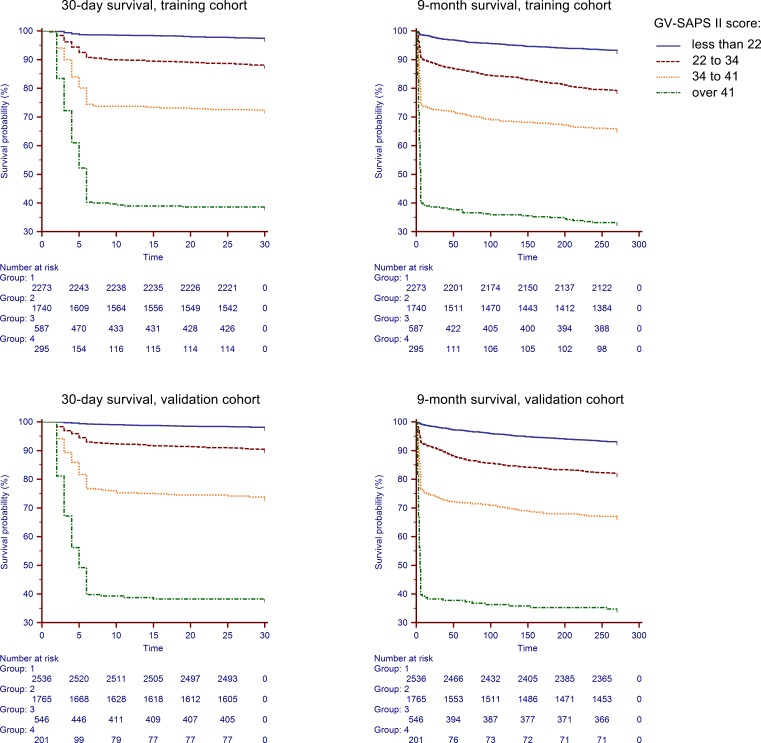
Survival distributions of different risk levels of the GV-SAPS II scoring system in the training and validation cohort.

To understand the survival distributions in different risk levels of the novel scoring system, we classified GV-SAPS II score into quartiles as follows: group 1 (< 22); group 2 (22 to 34); group 3 (34 to 41); group 4 (> 41). As shown in Fig 3, Kaplan-Meier curves indicate significantly worse outcomes in patients in higher score groups for both 30-day and 9-month mortality (all P< 0.001).

## Discussion

SAPS is one of many ICU scoring systems, which has been available since 1984 and designed to measure and predict the severity and prognosis of disease. The SAPS II score is calculated from 12 physiological measurements including age, heart rate, systolic blood pressure, temperature, GCS, mechanical ventilation or CPAP, PaO_2_, FiO_2_, urine output, blood urea nitrogen, sodium, potassium, bicarbonate, bilirubin, white blood cell, chronic diseases, type of admission. In this study, we constructed a modified SAPS II scoring system by adding glucose variability parameters (hyperglycemia, hypoglycemia, SD of blood glucose levels), named GV-SAPS II, for non-diabetic critically ill subjects. Although it was based on 30-day outcomes, we have demonstrated a prognostic value both in short-term and long-term mortality measurements using ROC analysis. In comparison with other standard scoring systems, GV-SAPS II performed significantly better with a higher auROC in both training and validation cohorts. Moreover, Kaplan-Meier survival curves showed that higher GV-SAPS II score groups were associated with a higher risk for death at 30 days and 9 months.

To our knowledge, this is the first modified prognostic scoring system that integrates glucose variability for non-diabetic critically ill patients. In previous studies, various physiological parameters were considered to build scoring systems, including serum glucose concentration. After adjusting for other parameters, the glucose level showed no significance prognostic capability [6,11]. In contrast, abnormal glucose levels have been demonstrated to represent an increased risk of mortality in several studies of critically ill patients [22,23,24,25]. The conclusion for these contradictory observations suggests that glucose variability, rather than serum glucose concentration has a crucial role in the mortality of critically ill patients [26,27,28,29]. From a clinical perspective, a single point serum glucose measurement can be easily influenced by a wide range of confounders, such as drug, diet, inflammation and physiological stress state. Therefore, it may not adequately reflect metabolic state in patients with critical illness. On the contrary, glucose variability may reveal dynamic changes of glucose levels, assessing the control of blood glucose. Moreover, the underlying mechanism for glucose variability has been reported to be associated with oxidative stress, neuronal damage, and blood coagulation activity [13,21]. In a vitro study, it has been demonstrated that acute fluctuations of glucose may be more detrimental to endothelial cell function than a constant abnormal level of glucose. This may contribute to increasing cardiovascular risk and reducing the homeostatic potential of the vasculature to accommodate perturbations in stress [30]. Thus it is feasible that glucose variability may play an important role in pathological processes associated with patients who are critically ill, suggesting that glucose variability should be considered as a part of the future development of prognostic models predicting patient mortality.

In this study, patients with diabetes were excluded from our study. Although glucose variability has been shown to be an independent risk factor in several mixed cohorts, previous studies have reported nonsignificant association between glucose variability and mortality of patients with diabetes [29,31]. In addition, it is a subject of debate as to whether hyperglycemia is an independent risk factor for patients with diabetes in ICU [29,32,33]. It has been proposed that these patients may become desensitized to rapid fluctuation of glucose levels, however, firm evidence is lacking and future research needs to establish specific models which may be applicable to patients with diabetes.

There are four main limitations of the present study. First, this is a single center cohort study and different conclusions may be reached using patient records from other centers, suggesting that a multicenter and prospective study are needed. Secondly, in order to ensure the accuracy of glucose variability, enough times of blood glucose test are needed. Thirdly, although SAPS II is still most widely used model, SAPS III has been available since 2005 [34] and a lack of surgery site information in the patient data base precluded a comparison of our model with SAPS III. Fourthly, due to the inconsistent pattern for glycemic metabolism, the diabetes patients have been excluded in our study. This may limit the scope of this scoring tool. Additionally, patients with impaired fasting glucose or impaired fasting glucose may be included in our cohort, which may have an impact on our prognostic system.

## Conclusions

We have constructed a modified prognostic scoring system that integrates glucose variability for non-diabetic patients who are critically ill. The GV-SAPS II scoring system was shown to have superior prognostic capability in study cohorts and may have utility as a scoring system for medical decision making and prognostic evaluation.

## Supporting Information

S1 File Minimal Data Set. (XLSX)

S1 Table STARD Checklist. (DOCX)

## Abbreviations

MIMIC-III: Multiparameter Intelligent Monitoring in Intensive Care database III
ICU: intensive care unit
SAPS: simplified acute physiology score
SOFA: sepsis-related organ failure assessment score
APACHE: acute physiology and chronic health evaluation
GCS: Glasgow Coma Scale
GV-SAPS II: Glucose Variability associated SAPS II Scoring System
SQL: structure query language
auROC: area under the curve of the receiver operator characteristic
SD: standard deviation
IQR: inter-quartile range
Glu_SD_: standard deviation of blood glucose levels
Glu_avg_: average of glucose levels
HR: hazard ratio
CI: confidence interva
GV: glucose variability
WBC: white blood cell
BUN: urea nitrogen
PO_2_: partial pressure of oxygen.
